# Expression of carboxyl terminus of Hsp70-interacting protein (CHIP) indicates poor prognosis in human gallbladder carcinoma

**DOI:** 10.3892/ol.2013.1138

**Published:** 2013-01-15

**Authors:** ZHE LONG LIANG, MEERAN KIM, SONG MEI HUANG, HYO JIN LEE, JIN-MAN KIM

**Affiliations:** 1 Departments of Pathology and Chungnam National University School of Medicine, Jung-Gu, Daejeon 301-131, Republic of Korea; 2 Internal Medicine, Chungnam National University School of Medicine, Jung-Gu, Daejeon 301-131, Republic of Korea

**Keywords:** carboxyl terminus of Hsp70-interacting protein, gallbladder carcinoma, prognosis, survival

## Abstract

Gallbladder carcinoma (GBC) is a lethal neoplasm, and new prognostic markers are required. Deregulation of E3 ligases contributes to cancer development and is associated with poor prognosis. Carboxyl terminus of heat shock protein 70-interacting protein (CHIP) is a U-box-type E3 ubiquitin ligase, the role of which has not been evaluated in GBC. Therefore, the present study investigated CHIP expression in GBC and its prognostic significance. In the present study, CHIP expression was measured in 78 tumor specimens of GBC by immunohistochemistry and the correlation between CHIP expression and clinicopathological factors was analyzed. Of the tumor specimens, 26.9% showed high staining intensity [the CHIP high expression group (HEG)]. The CHIP-HEG was not associated with other common clinicopathological parameters, including T stage, and lymph node and distant metastases. CHIP-HEG patients had a significantly worse prognosis than patients with low CHIP expression with median cancer-specific survival times of 8.0 months (range, 1–34 months) and 13.0 months (range, 1–110 months), respectively (P=0.023). Multivariate analyses showed that CHIP expression was close to being an independent risk factor for predicting patient survival. CHIP expression may be associated with a poor prognosis in GBC. Since CHIP is not associated with other clinicopathological prognostic factors, it may serve as an ideal molecular marker for predicting patient outcomes.

## Introduction

Gallbladder carcinoma (GBC) is a relatively uncommon neoplasm in the majority of countries and its incidence rate shows marked geographic and ethnic variation. It is up to three times more common in females compared with males in almost all populations. The highest incidences in the world are among women from Chile (27/100,000), Poland (14/100,000), India (10/100,000), Japan (7/100,000) and Israel (5/100,000). In the United States and the United Kingdom, the incidence is <2/100,000 (1–3). GBC is a highly lethal disease since it is usually diagnosed at an advanced stage (4). The 5-year survival rate of patients with GBC is ∼10–30% despite surgical resection (5–7). Moreover, the majority of patients have frequent recurrences following surgery and unsatisfactory results following chemotherapy or radiotherapy (8). Several prognostic models have been designed to identify patients with a high risk of disease progression following cholecystectomy, and features such as grade, depth of wall infiltration and lymph node metastasis have been determined to be classic clinicopathological prognostic factors (9). In addition, certain molecular biomarkers have been identified including cyclooxygenase-2 (Cox2) and hypoxia-inducible factor (HIF) (10–21). However, the majority of these molecular and genetic factors are not markedly associated with GBC. Therefore, it would be useful to identify new molecular markers, that may be associated with prognosis and used as therapeutic targets.

Carboxyl-terminus of heat shock protein 70-interacting protein (CHIP) is a well-described U-box-type E3 ubiqutin ligase that induces ubiquitination and proteasomal degradation of its substrates, which include several tumor-related proteins (22–26). CHIP participates in the degradation of p53, a common tumor suppressor protein frequently mutated in cancers (27,28). The stress-dependent death domain-associated protein (Daxx)-CHIP interaction also suppresses p53 apoptotic pathways (29). Through the formation of the heat shock protein 70 (HSP70)/CHIP/apoptosis signal-regulating kinase 1 (ASK1) complex, HSP70 promotes ASK1 proteasomal degradation and prevents tumor necrosis factor-α (TNF-α)-induced cell apoptosis (30). In addition, CHIP is a negative regulator of forkhead class O1 transcription factor (FoxO1) activity through ubiquitin-mediated degradation, thus promoting cell survival (31). Xu *et al* demonstrated that CHIP contributes to the tumorigenesis of malignant gliomas by regulating survivin (32). These data suggest that CHIP may be significant in cancer by regulating tumor-related proteins. However, the clinical relevance of CHIP in GBC has not been investigated. The present study analyzed the expression of CHIP in tumor specimens from patients who underwent surgical treatment of GBC and investigated the association between CHIP expression and clinicopathological features, as well as patient survival.

## Materials and methods

### Patients and tumor samples

Tumor samples from 78 consecutive patients who underwent cholecystectomy for GBC at the Chungnam National University Hospital from 1999 to 2010 were investigated. Clinicopathological data were obtained by reviewing medical records. The patient population included 38 males and 40 females who ranged in age from 25 to 87 years (median, 68 years). All tumors were diagnosed as adenocarcinoma and defined as primary tumors arising from the gallbladder. The T classification was defined according to 2002 American Joint Committee on Cancer criteria. Tumor samples were collected from tissue blocks used for routine pathological examination. All patients signed informed consent for therapy, as well as for subsequent tissue studies, which had received prior approval by the local ethics committee.

### Histological grading

The GBC specimens were examined by routine hematoxylin and eosin staining. The specimens were graded into well- (G1), moderately (G2) and poorly differentiated (G3) and undifferentiated (G4) adenocarcinoma according to the World Health Organization classification. Eight cases were well differentiated, 46 were moderately differentiated, 21 were poorly differentiated and three were undifferentiated.

### Tissue microarray (TMA) construction

TMAs were constructed from archival, original formalin-fixed, paraffin-embedded tissue blocks from 78 patients with GBC. For each tumor, a representative tumor area was carefully selected from a hematoxylin and eosin stained section of a donor block. Each case was represented by two 2-mm-diameter core cylinders from tumors which were obtained using an automated tissue array (UNITMA, Seoul, Korea). TMA blocks containing a total of 156 cylinders were constructed from these samples.

### Immunohistochemistry (IHC)

The expression of CHIP was analyzed by IHC on paraffin-embedded tissue sections from the GBC samples. Sections (3-μm thick) from the paraffin blocks were used for IHC with the rabbit EnVision-HRP detection system (Dako, Carpinteria, CA, USA). A polyclonal rabbit CHIP antibody (AP6413a; ABGENT, San Diego CA, USA) was used for IHC. Following deparaffinization and antigen retrieval with a pressure cooker in 10 mM sodium citrate buffer (pH 6.0) at full power for 4 min, the tissue sections were treated with 3% hydrogen peroxide for 10 min. The primary antibody was diluted (1:100) with background-reducing diluents (Dako) and incubated overnight at 4°C in a humid chamber. The slides were then incubated with the EnVision reagent for 30 min, sequentially incubated with DAB chromogen for 5 min, counterstained with Meyer’s hematoxylin and mounted. Careful rinsing with several changes of 0.3% Tween-20 in TBS buffer was performed between each step. For the IHC negative control, the primary antibody was excluded.

### Evaluation of immunostaining

The level of CHIP expression in each sample was evaluated by two independent pathologists (J.M. Kim and M.R. Kim) who were blinded to the patients’ clinicopathological details. The IHC staining was categorized according to a scoring method in which the tumors were classified into four grades based on the staining intensity: (0, no staining; +1, low staining intensity; +2, intermediate staining intensity; and +3, high staining intensity). In cases of heterogeneous staining within the samples, the higher score was selected if >50% of the cells exhibited the higher staining intensity. For all patients, the scores from the two tumor cores from the same patient were averaged to obtain a mean score. Cases with staining intensity scores of 0, +1 and +2 were included in the CHIP low-expression group (LEG), whereas those with staining intensity scores of +3 were included in the CHIP high-expression group (HEG) for all analyses.

### Statistical analysis

Group comparisons of categorical variables were evaluated using the χ^2^ test or the linear-by-linear association test. Cancer-specific survival was defined from the date of surgery to the date of mortality by GBC. Survival curves were plotted using the Kaplan-Meier method and analyzed using the log-rank test. Cox’s proportional hazards model was used to identify prognostic factors for survival. For all analyses, P<0.05 was considered to indicate a statistically significant difference. All statistical analyses were performed using the SPSS version 17.0 statistical software (SPSS Inc., Chicago, IL, USA).

## Results

### Immunohistochemical analysis of CHIP expression in GBC

**Figure 1:**
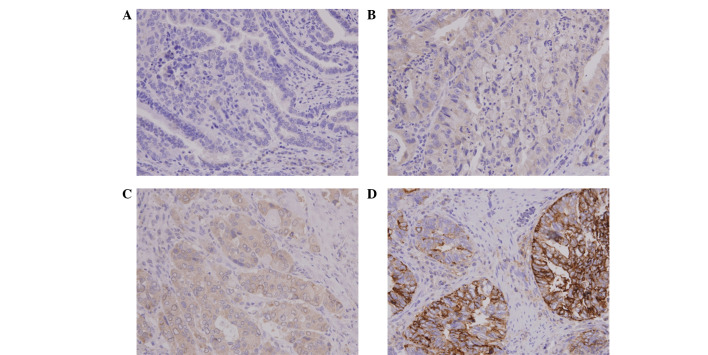
Representative photomicrographs of immunohistochemical staining for CHIP in human gallbladder cancer tissues. (A) No staining intensity, (B) weak staining intensity, (C) intermediate staining intensity and (D) strong staining intensity. Original magnification, x400. CHIP, carboxyl terminus of Hsp70-interacting protein.

CHIP expression was analyzed by IHC analysis of the tumor specimens obtained from 78 patients with GBC. CHIP staining varied by intensity and location. It was predominantly located in the cytoplasm of the GBC cells, but also appeared in the nucleus or cell membrane in certain cells (Fig. 1). Next, the CHIP expression levels were analyzed by determining the intensity of the positively stained tumor cells. A total of 21 cases (26.9%) had +3 staining intensity (CHIP-HEG) and 57 cases had a lower staining intensity (CHIP-LEG), specifically +2 (23 cases), +1 (31 cases) or 0 (3 cases).

### Correlation between CHIP expression and cliniocopathological factors

The correlations between CHIP expression and various clinicopathological factors known to affect the prognosis of patients with GBC were analyzed. The results are presented in Table I. No significant differences were observed with regard to age and gender between the CHIP-LEG and CHIP-HEG patients. In addition, other clinicopathological factors, such as pathological T stage, lymph node and distant metastases, stage, differentiation, perineural invasion and lymphatic invasion were not significantly associated with CHIP expression.

### Correlation between CHIP expression and survival

**Figure 2:**
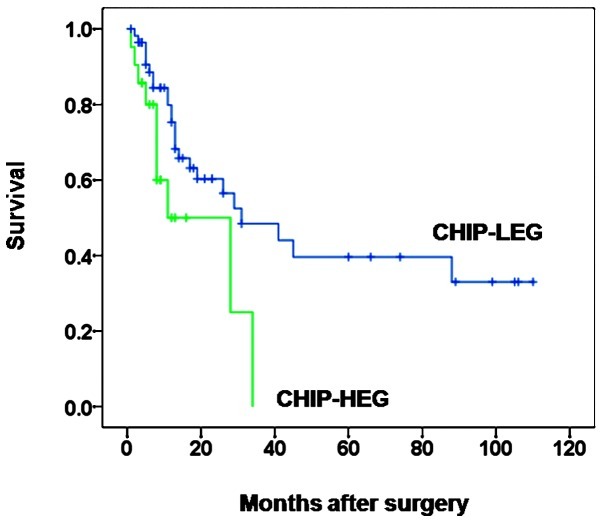
Survival curve according to CHIP expression in patients with gall-bladder carcinoma (P=0.023). CHIP, carboxyl terminus of Hsp70-interacting protein; LEG, low-expression group; HEG, high-expression group.

To determine the clinical utility of CHIP expression with regard to the GBC prognosis, the association between CHIP expression and patient survival was investigated. Survival curves according to CHIP expression are shown in Fig. 2. The median cancer-specific survival rates were 8.0 months (range, 1–34 months) and 13.0 months (range, 1–110 months) in patients with CHIP-LEG and -HEG tumors, respectively (P=0.023). Next, univariate analyses were performed to estimate the clinical significance of various parameters that may affect survival in patients with GBC. As shown in Table II, pathological T stage (P=0.002), stage (P=0.002), differentiation (P=0.047), lymphatic invasion (P=0.027) and CHIP expression (P=0.029) were statistically significant risk factors affecting the cancer-specific survival of patients with GBC. To determine the independent prognostic effects of these various factors, multivariate analyses using Cox’s proportional hazards model were performed. The model revealed that none of these factors were independent risk factors for predicting short-term cancer-specific survival, although CHIP expression (hazard ratio, 2.221; 95% confidence interval, 0.984–5.016; P=0.055) was close to being a significant independent risk factor for predicting patient survival (Table III).

## Discussion

The present study is the first to demonstrate that CHIP expression varied among GBC samples and that there was a significant difference in cancer-specific survival between CHIP-LEG and CHIP-HEG groups. Since CHIP was not associated with other clinicopathological prognostic parameters, such as pathological T stage, nodal and distant metastases, stage, differentiation, perineural invasion and lymphatic invasion, it may be an ideal complementary molecular marker for predicting patient outcomes in GBC. Univariate analyses clearly demonstrated that CHIP expression was a statistically significant risk factor for the cancer-specific survival of patients with GBC and multivariate analyses showed that it was close to being an independent risk factor. The statistically significant effect of CHIP expression was more significant than that of the various clinicopathological parameters that are widely used at present, suggesting that CHIP expression may be a useful marker for predicting patient survival.

Several prognostic molecular markers for GBC have been described previously. Mutations in p53 and v-Ki-ras2 Kirsten rat sarcoma viral oncogene homolog (K-ras), cycle-related proteins p16 and p21, Cox2, vascular endothelial growth factor (VEGF), c-erb-B2 (HER-2/neu), inducible nitric oxide synthase (iNOS) and adhesion molecules (E-cadherin, β-catenin, CD54 and CD44) have been studied (10–20). We previously reported that L1 cell adhesion molecule expression is a novel independent prognostic factor that indicates a poor prognosis for patients with gallbladder carcinoma (21). The deregulation of E3 ligases contributes to cancer development and their overexpression is often associated with poor prognosis, as has been shown in studies of inhibitor of apoptosis protein (IAP)-family genes (33), murine double minute 2 (Mdm2) (34), Casitas B-lineage lymphoma (CBL)-family proteins (35) and anaphase promoting complex (APC) (36). Li *et al* reported that the overexpression of Skp2, an Skp1-Cullin-F-box protein (SCF) ubiquitin ligase-related protein, was significantly correlated with unfavorable clinicopathological parameters and short-term survival (37). Furthermore, certain E3 ubiquitin ligases have emerged as therapeutic targets for cancer (38,39). In the present study, it was observed that CHIP, a member of the E3 ubiquitin ligase family, was associated with poor prognosis for GBC. These results provide further support for E3 ligases as biological markers for GBC.

The pathogenic mechanism of CHIP expression in human malignancy is not yet clear and a number of studies have suggested that CHIP may have opposing roles in different cancers (22–26,40). CHIP suppresses tumor progression in human breast cancer by inhibiting oncogenic pathways and CHIP levels are negatively correlated with the malignancy of human breast tumor tissues. The anchorage-independent growth and invasiveness of CHIP-knockout cells is significantly elevated due to the increased expression of B-cell CLL/lymphoma 2 (Bcl2), protein kinase B (Akt)1, small mothers against decapentaplegic (Smad) and Twist, a transcription factor. Proteomic analysis identified the transcriptional co-activator steroid receptor coactivator 3 (SRC-3) as a direct target for ubiquitylation and degradation by CHIP (40). Another study noted that the overexpression of CHIP inhibited the lung cancer cell growth and invasion mediated by Met (the receptor for hepatocyte growth factor) (26). By contrast, Xu *et al*(32) showed that CHIP contributed to the tumorigenesis of human gliomas by regulating survivin. The authors also observed that CHIP expression in glioma samples was associated with tumor grades, with more marked staining in high-grade gliomas compared with low-grade gliomas. A knockdown of CHIP expression suppressed the proliferation and colony formation of glioma cells, while the overexpression of CHIP resulted in enhanced proliferation and colony formation *in vitro*. An intratumoral injection of CHIP RNA interference (RNAi) lentivirus significantly delayed tumor growth and was associated with decreased mRNA and protein levels of survivin in a nude mouse xenograft model, while CHIP overexpression resulted in enhanced tumor growth and increased the mRNA and protein levels of survivin *in vivo*(32). Collectively, whether CHIP contributes to tumor progression or tumor suppression in various human cancers remains unclear, suggesting the necessity of further extensive investigation of its role in tumorigenesis.

In conclusion, the present results indicate that, as a member of the E3 ubiquitin ligase family, CHIP was differentially expressed in GBC and higher expression levels were associated with poor prognosis in patients who were surgically treated for GBC, suggesting that it may be a useful molecular marker in GBC. However, the present study was limited by its small sample size and retrospective nature. Further prospective investigations with a large number of patients would allow an improved understanding of the important role of CHIP in GBC progression. Molecular studies are also required to elucidate the pathogenic mechanism of CHIP’s involvement in GBC.

## Figures and Tables

**Table I:** Association of CHIP expression with clinicopathological characteristics of gallbladder carcinoma.

		CHIP		
Variable	Total n=78	LEG (n=57) %	HEG (n=21) %	P-value
Age (years)				0.676^a^
<65	22	17 (29.8)	5 (23.8)	
≥65	56	40 (70.2)	16 (76.2)	
Gender				0.157^a^
Male	38	25 (43.9)	13 (61.9)	
Female	40	32 (56.1)	8 (38.1)	
Pathological T stage				0.675^b^
1	15	9 (15.8)	6 (28.6)	
2	35	28 (49.1)	7 (33.3)	
3	25	18 (31.6)	7 (33.3)	
4	3	2 (3.5)	1 (4.8)	
Nodal metastasis				0.155^a^
Absent	56	38 (66.7)	18 (85.7)	
Present	22	19 (33.3)	3 (14.3)	
Distant metastasis				0.559^a^
Absent	75	54 (94.7%)	21 (100)	
Present	3	3 (5.3%)	0 (0)	
Stage				0.623^a^
I	41	29 (50.9)	12 (57.1)	
II–IV	37	28 (49.1)	9 (42.9)	
Differentiation				0.432^b^
G1	8	4 (7.0)	4 (19.0)	
G2	46	36 (63.2)	10 (47.6)	
G3	21	14 (24.6)	7 (33.3)	
G4	3	3 (5.3)	0 (0)	
Perineural invasion				0.596^a^
Absent	37	26 (45.6)	11 (52.4)	
Present	41	31 (54.4)	10 (47.6)	
Lymphatic invasion				0.353^a^
Absent	27	18 (31.6)	9 (42.9)	
Present	51	39 (68.4)	12 (57.1)	

CHIP, carboxyl terminus of Hsp70-interacting protein; LEG, low expression group; HEG, high expression group.

a P values were calculated by pairwise comparisons from χ^2^ test or Fisher’s exact test.

b P values were calculated by comparisons of four groups from linear-by-linear associations.

**Table II:** Univariate analysis of the association of prognosis with clinicopatholocal parameters and CHIP expression in patients with gallbladder carcinoma.

Variables	Hazard ratio	95% confidence interval	P-value
Age (years, ≥65 vs. <60)	2.064	0.896–4.756	0.089
Gender (male vs. female)	1.579	0.799–3.118	0.188
Pathological T stage (T3/4 vs. T1/2)	3.028	1.525–6.014	0.002
Nodal metastasis (yes vs. no)	1.618	0.762–3.434	0.210
Distant metastasis (yes vs. no)	1.965	0.461–8.368	0.361
Stage (II–IV vs. I)	3.212	1.552–6.648	0.002
Differentiation (G3/4 vs. G1/2)	1.990	1.008–3.928	0.047
Perineural invasion (yes vs. no)	1.878	0.872–4.043	0.107
Lymphatic invasion (yes vs. no)	2.938	1.133–7.613	0.027
CHIP (HEG vs. LEG)	2.373	1.093–5.152	0.029

CHIP, carboxyl terminus of Hsp70-interacting protein; LEG, low expression group; HEG, high expression group.

**Table III:** Multivariate analysis of the association of prognosis with clinicopathological parameters and CHIP expression in patients with gallbladder carcinoma.

Variables	Hazard ratio	95% confidence interval	P-value
Pathological T stage (T3/4 vs. T1/2)	1.421	0.406–4.974	0.582
Stage (II–IV vs. I)	2.075	0.542–7.951	0.287
Differentiation (G3/4 vs. G1/2)	1.499	0.714–3.149	0.285
Lymphatic invasion (yes vs. no)	1.681	0.581–4.861	0.338
CHIP (HEG vs. LEG)	2.221	0.984–5.016	0.055

CHIP, carboxyl terminus of Hsp70-interacting protein; LEG, low expression group; HEG, high expression group.
